# ‘Oxygen-based antiseptics’ potential in the management of peri-implant mucositis

**DOI:** 10.17179/excli2025-8491

**Published:** 2025-07-25

**Authors:** Thalles Yurgen Balduino, André Felipe dos Santos Teles, Gabriel Leonardo Magrin, Marco Aurélio Bianchini

**Affiliations:** 1 Department of Dentistry, Center for Education and Research on Dental Implants (CEPID), Federal University of Santa Catarina (UFSC), 88040-900 Florianópolis, SC, Brazil

## ⁯

The growing global demand for dental implants, mainly driven by their high survival rates and patient satisfaction, has been accompanied by a rise in the prevalence of biological complications, particularly peri-implant diseases: mucositis and peri-implantitis. These conditions present a growing challenge in the contemporary clinical practice, especially due to the fact that mucositis, if not managed properly, can eventually lead to peri-implantitis, a more severe condition that involves the bone around the implant, resulting in bone defects, soft-tissue dehiscences and ultimately implant loss (Galárraga-Vinueza et al., 2020[4]).

Peri-implant mucositis is characterized by inflammation of the peri-implant soft tissues, without bone loss (Heitz-Mayfield, 2024[5]). Its etiopathogenesis involves biofilm formation and the consequent imbalance of the resident microbiota, favoring gram-negative anaerobic bacteria, leading to local changes in pH and proteolytic metabolism, creating a hostile environment for healing and perpetuating inflammation (Lindhe and Lang, 2018[7]). Studies indicate a high prevalence of peri-implant mucositis amongst patients rehabilitated with dental implants, with rates ranging from 19 % to 65 % (Derks and Tomasi, 2015[1]).

Despite its reversible characteristics, the peri-implant mucositis represents a critical stage of the inflammatory process of the peri-implant diseases, being considered a precursor of peri-implantitis if not managed accordingly (Heitz-Mayfield, 2024[5]). It is important to highlight that peri-implant mucositis usually presents a positive response to non-surgical treatment, which consists of mechanical removal of the biofilm associated with a chemical treatment (Ramanauskaite et al., 2021[8]).

In this context, oxygenating anti-plaque agents have emerged as a smart alternative to broad-spectrum antimicrobial agents. Their use has shown various benefits, particularly due to their efficacy in reducing the microbial load of the biofilm and delaying the anaerobic bacteria colonization, without compromising the resident microbiota responsible for maintaining an ecological balance (Vasthavi et al.,2020[10]).

The presence of active oxygen in the peri-implant environment stimulates the cellular metabolism and energy production, favoring the angiogenesis process and local revascularization (Fernandez y Mostajo et al., 2014[3]). The vascular neogenesis is crucial for the support and regeneration of the soft tissues, especially for the fibroblast development, whose adequate oxygen levels are determinant, such for speed as to quality of the vascular growth, as shown in supplementary Figure 1 (Juliana and Tarek, 2022[6]; Fernandez y Mostajo et al., 2014[3]).

*In vitro* and clinical studies have demonstrated that biofilms exposed to active oxygen exhibit significant reductions in microbial load, decreasing up to 40 % in colony-forming units, compared to negative controls (Fernandez y Mostajo et al., 2017[2]). Moreover, active oxygen has proven more effective than chlorhexidine in reducing red complex pathogens (Shibli et al., 2021[9]). This additional antimicrobial mechanism involves increasing the lactic acid release, improving phagocytosis, and removing necrotic debris, thus supporting the restoration of ecological balance and tissue integrity (Fernandez y Mostajo et al., 2017[2]).

The clinical relevance of oral antiseptics lies not only in the immediate therapeutic effect but also in the duration of their efficacy. Regulatory agencies such as the American Dental Association (ADA) require that products with biofilm-control effects containing active oxygen undergo evaluation for at least a period of four weeks, with follow-up assessments extending up to six months (Fernandez y Mostajo et al., 2017[2]).

These findings highlight the potential of oxygen-based antiseptics as a promising alternative in the management of peri-implant mucositis. Considering the limitations of current treatment protocols and the favorable biological effects of oxygen, there is a clear need for further clinical studies to deepen this approach and enhance patient care outcomes.

## Conflict of interest

The authors declare that they have no conflict of interest.

## Supplementary Material

Supplementary information
