# Supplementary material for: ‘Oxygen-based antiseptics’ potential in the management of peri-implant mucositis
Source: EXCLI J. 2025 Jul 25;24:955–6. doi: 10.17179/excli2025-8491 (PMC12635826; doi:10.17179/excli2025-8491)
Supplement: Supplementary information [file EXCLI-24-955-s-001.pdf]

EXCLI Journal 2025;24:955-956 – ISSN 1611-2156
Received: April 29, 2025, accepted: June 12, 2025, published: July 25, 2025

Supplementary information to:

Letter to the editor:

'OXYGEN-BASED ANTISEPTICS' POTENTIAL IN THE MANAGEMENT OF PERI-IMPLANT MUCOSITIS

Thalles Yurgen Balduino*, André Felipe dos Santos Teles, Gabriel Leonardo Magrin, Marco Aurélio Bianchini

Department of Dentistry, Center for Education and Research on Dental Implants (CEPID), Federal University of Santa Catarina (UFSC), 88040-900 Florianópolis, SC, Brazil

* **Corresponding author:** Thalles Yurgen Balduino, Department of Dentistry, Center for Education and Research on Dental Implants (CEPID), Federal University of Santa Catarina (UFSC), 88040-900 Florianópolis, SC, Brazil. E-mail: thallesbalduino@hotmail.com

https://dx.doi.org/10.17179/excli2025-8491

This is an Open Access article distributed under the terms of the Creative Commons Attribution License (https://creativecommons.org/licenses/by/4.0/).

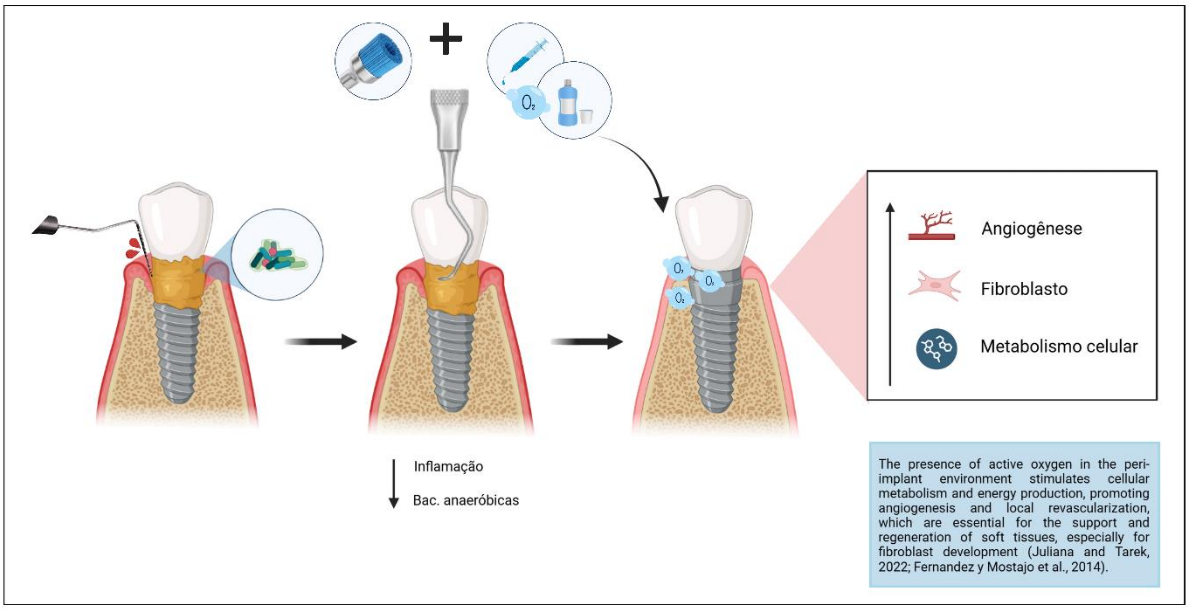

Supplementary Figure 1: Stimulation of peri-implant soft tissue regeneration with active oxygen application after mechanical debridement

Notes: Active oxygen promotes angiogenesis, enhances cellular metabolism, and stimulates fibroblast proliferation, supporting the healing of inflamed soft tissues.
